# Dr. Chisholm

**Published:** 1825-03

**Authors:** 


We have likewise to regret the death of another distinguished medical officer, Dr. CHISHOLM, who died lately at Geneva, at an advanced age. Dr. C. was attached to the ordnance department of the army, and his principal services were in the West Indies. He took an active part in the investigations connected with the Yellow Fever; his works are before the public, and must be known to the readers of this Journal, having been at different times favourably noticed: they are distinguished by zeal, candour, and good sense.

